# Scoliosis in Duchenne muscular dystrophy children is fully reducible in the initial stage, and becomes structural over time

**DOI:** 10.1186/s12891-019-2661-6

**Published:** 2019-06-07

**Authors:** Young-Ah Choi, Hyung-Ik Shin, Hyun Iee Shin

**Affiliations:** 1 Department of Rehabilitation Medicine, Incheon St. Mary’s Hospital 59, Dongsu-ro, Bupyeong-gu, Incheon, 21431 Korea; 2 Seoul National University Hospital, Department of Rehabilitation Medicine, 101 Daehak-ro, Jongno-gu, Seoul, 110-744 Korea

**Keywords:** Duchenne muscular dystrophy, Flexibility, Nonstructural scoliosis, Structural scoliosis, Pelvic obliquity, Neuromuscular scoliosis

## Abstract

**Background:**

Patients with Duchenne muscular dystrophy (DMD) often develop scoliosis that progresses rapidly after loss of ambulation. Management of scoliosis is crucial because it affects both life expectancy and quality of life of patients with DMD. Spinal orthosis attempts to prevent or delay scoliosis using spinal support at three points of the controlling mechanism; the curve should be flattened by the pressure. Therefore, it is assumed that spine flexibility could be a significant influencing factor for the effectiveness of braces. Hence, we attempted to investigate the flexibility of scoliosis in non-ambulant patients with DMD.

**Methods:**

We reviewed the medical records of 273 boys who were genetically identified as having DMD, and finally, 50 boys with serial records of radiographs after loss of ambulation were finally enrolled. And among them, only 31 patients developed scoliosis. Spine radiographs in sitting and supine positions were also reviewed to obtain Cobb angle, curve flexibility, and pelvic obliquity. Flexibilities (%) were calculated by the difference in angles between the sitting and supine positions divided by the angle at the sitting position, multiplied by 100.

**Results:**

Among 31 boys who had scoliosis, all but 2 boys with curves went through a sequential course of 1) no scoliosis, 2) nonstructural scoliosis, when scoliosis was only measurable in the sitting position, and 3) structural scoliosis, when scoliosis was also detectable in the supine position. Flexibility decreased each year after detection of scoliosis in those who developed scoliosis the first year, from 75.5 ± 5.0% to 57.1 ± 10.5% and to 49.1 ± 10.0% (mean ± standard deviation). Spinal flexibility was significantly correlated with curve magnitude of scoliosis in both sitting and supine position (*p* < 0.05, respectively).

**Conclusions:**

There is a period of fully reducible curve in DMD patients at the initial stage of scoliosis. Afterward, as spinal curve progresses, flexibility decreases over time. To detect the scoliosis when the curve is fully reducible, scoliosis curve in DMD patients should be evaluated dynamically, including radiographs of at least in two different positions.

## Background

Scoliosis is a frequent complication of Duchenne muscular dystrophy (DMD) that progresses rapidly, in the non-ambulatory stage of the disease [1–6]. Pelvic obliquity is also thought to be a mechanism of compensation for scoliosis [7]. These deformities in the musculoskeletal system together make sitting difficult, limiting the use of upper extremities and hampering activities of daily living. When scoliosis progresses, rib impingement onto the ilium may occur, causing pain and making hygiene difficult [8]. It is crucial to prevent scoliosis as it affects other organ systems.

After loss of ambulation, rapid progression of spinal deformity leads to a deterioration in pulmonary function [3, 5, 6]. Kurz et al. reported that with 10 degrees of thoracic curve progression, functional vital capacity decreased by 4% [9]. According to Hsu et al., in DMD patients whose spinal curves exceeded 40 degrees, vital capacity diminished by 12 to 16% [10]. Therefore, it is important to prevent, or delay spinal deformity as it leads to compromise of respiratory function.

Spinal orthosis attempts to prevent or delay scoliosis using spinal support at three points of the controlling mechanism; the lateral curve should be flattened by the pressure. Therefore, it is assumed that spine flexibility or reducibility is a significant influencing factor for the effectiveness of braces [11, 12]. Information regarding curve flexibility helps establish a strategy for brace application to manage scoliosis. If there is sufficient flexibility, the effectiveness of bracing therapy is expected.

Nevertheless, there have been only a few reports investigating spine flexibility in this patient group [13].

Therefore, this study is to investigate the curve flexibility of scoliosis for 2 years after loss of walking ability in children with DMD.

## Methods

### Subjects

Medical records and radiographs of 273 boys diagnosed with DMD who visited the pediatric rehabilitation department between March of 2017 and February of 2018 were reviewed. Ethical approval was obtained from the Institutional Review Board (IRB No. 1804–169-942). DMD diagnosis had been established using a dystrophin gene study. The genetic test methods used to identify dystrophin mutations included multiplex polymerase chain reaction and direct sequencing (Xp21.2-p21.1, exons 1–79). If deletion/duplication testing results were negative, then dystrophin gene sequencing was performed to search for point mutations or small deletions/insertions. All enrolled DMD pediatric patients were taking deflazacort (0.9 mg/kg) every other day, according to international consensus, at the pediatric department in the same hospital after a partial Gower sign had been observed [4].

Inclusion criteria were as follows: (1) time points of the ambulation loss were charted; (2) 2-year records of whole spine radiographs both in supine and sitting positions were preserved with (3) the first follow-up radiography was performed less than 1 year after the onset of ambulation loss.

### Review of medical records

Patients with DMD in our hospital had regular outpatient follow-up at 12-month intervals when ambulatory and 6 months after the loss of ambulation [14]. Whole spine radiographs were taken in the sitting and supine positions, and ambulatory functions were charted in Vignos scales at each outpatient follow-up. The onset of ambulation loss was defined when the charted scale value exceeded or equaled grade 8, when patients were able to stand with long leg braces, but were unable to walk even with assistance [15].

### Evaluation of scoliosis

The authors designated the patients who had no scoliosis both in sitting and supine positions as having “no scoliosis;” those who only had scoliosis in sitting position but not in supine position were designated as having “nonstructural scoliosis;” and those who had scoliosis both in sitting and supine positions were designated as having “structural scoliosis” [11].

### Cobb angle and pelvic obliquity measurement

Postero-anterior radiographs of selected patients were used in this study. To improve intraobserver reliability, the measurements were taken by a single well-trained physician.

The image field in the cranio-caudal direction ranged from the occiput to the acetabula. Cobb angle of more than 10° was considered significant [3, 16, 17]. Cobb angles were retrospectively measured by a single observer. The most oblique cranial and caudal end vertebrae were marked, and lines were drawn through the endplates of each vertebra, and the angles between them were measured [18]. As measurement error of Cobb angle results from errors in selecting the end vertebrae [18, 19], initially selected end vertebrae were marked to be used for serial measurements to reduce measurement error.

The horizontal pelvic obliquity method measures angle between the line of most proximal iliac crests and the parallel line to the bottom of the radiograph. This angle is largely influenced by the patient’s position [20]. Pelvic obliquity more than 5° was considered significant [7, 21]. This horizontal pelvic obliquity measurement is associated with the least interobserver and intraobserver variability [20, 22]. To minimize these errors, the patients were confirmed to be in the maximal and appropriate position and were well-fitted to the frame when taking images.

### Evaluation of flexibility

Scoliosis curve flexibility was assessed by comparing the Cobb angle values in the supine position (gravity eliminated posture) and those in the sitting position (increase in the curve with gravity). Cobb angle and pelvic obliquity in each position and flexibility were analyzed at the time when scoliosis was first detected after ambulation loss, 1 year after scoliosis detection, and 2 years after scoliosis detection. The flexibility of the spine curve at each year was calculated as below [12, 23].

$$ Flexibility\ \left(\%\right)=\frac{Cobb\ angle\  at\  sitting- Cobb\ angle\  at\  supine\ position}{Cobb\ angle\  at\  sitting}\times 100 $$

For the supine position, the hands were placed by the patient’s side, and patients were instructed to lie down facing up on a scanning couch and then to straighten their trunk and legs maximally [24]. In the sitting position, patients were instructed to sit on a chair with a panel on the back, with the hip to be placed appropriately on the chair. They were asked to lie back maximally to eliminate tilts. The patients were instructed to hold handles on both sides during radiography. If they were unable to hold the handles, they were simply asked to lay their hands on the handles.

### Statistical analysis

The demographic characteristics and measurements of the participants were classified by scoliosis development. To analyze changes in flexibilities in the series of radiographs, repeated measure analysis of variance was used. To evaluate the relationships among each index, Spearman’s correlation tests were used. All data were statistically analyzed using the Statistical Package for Social Sciences for Windows ver. 17.0 (SPSS Inc., Chicago, IL, USA).

## Results

### Subjects

**Fig. 1:**
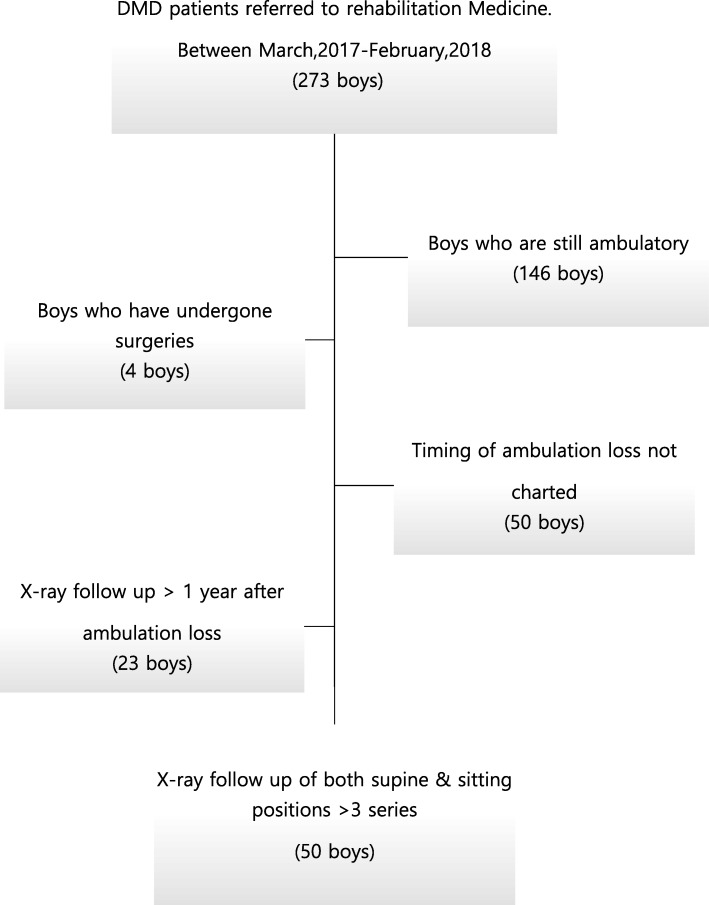
Flowchart of subject enrollment

Among 273 boys, 146 boys who were still ambulatory were excluded. In the remaining boys, 4 who had undergone spine surgeries during the follow-up period were also excluded. After excluding those without relevant medical information and radiologic records, 50 boys remained for analysis (Fig. 1). There were 31 boys who developed scoliosis during follow up period. Characteristics of participants according to scoliosis development is shown in Table 1. Age of boys at ambulation loss was 13.1 ± 2.6 years There were no significant difference between two groups except pulmonary function (*p* < 0.05). Among those who were included for analysis, no patient was prescribed for spinal orthosis.

**Table 1:** Demographic and clinical characteristics of participants

		Scoliosis development	No scoliosis	*P* value	Total
Number of boys		31	19	–	50
Age (year)		13.6 ± 2.5	12.4 ± 2.6	0.08	13.14 ± 2.6
Height (cm)		149.4 ± 25.5	142.9 ± 34.3	0.66	146.9 ± 25.6
Body weight (kg)		49.1 ± 16.5	45.9 ± 12.1	0.36	47.9 ± 14.9
Functional assessment	Brooke scale	2.5 ± 1.7	2.0 ± 1.0	0.31	2.3 ± 1.5
	Vignos scale	8.4 ± 0.6	8.3 ± 0.5	0.43	8.3 ± 0.5
Pulmonary function	FVC % of predicted (%)	74.2 ± 12.5	84.5 ± 15.2	0.03	78.2 ± 14.7

Values are presented as mean ± standard deviation. Comparison between groups was by Mann-Whitney test. FVC; Functional vital capacity

### Development of scoliosis

The 50 boys had 2 years of annual follow-up radiographs available from the onset of ambulation loss. During this follow-up period, 19 boys fell under the category of no scoliosis, and 31 boys developed either nonstructural or structural scoliosis. At the first follow-up after ambulation loss, 12 boys developed scoliosis, and another 13 boys developed scoliosis the following year. At the last year of follow-up, 6 more patients were found to have scoliosis (Table 2). Cobb angle increased each year after the ambulation loss both in sitting and supine positions (Table 2).

**Table 2:** Cobb angle of subjects

	Time 1			Time 2			Time 3		
Number of boys	Cobb angle(°)		Flexibility (%)	Cobb angle(°)		Flexibility (%)	Cobb angle(°)		Flexibility (%)
	sitting	supine		sitting	supine		sitting	supine	
12^a^	23.0 ± 5.6	10.8 ± 6.6	75.5 ± 5.0	30.9 ± 5.9	18.2 ± 6.6	57.1 ± 10.5	34.4 ± 4.4	22.2 ± 6.3	49.1 ± 10.0
25^a^	11.1 ± 3.5	5.2 ± 3.3	60.7 ± 6.5	27.2 ± 3.2	13.7 ± 3.6	36.2 ± 9.2			
31	22.4 ± 2.8	8.2 ± 3.0	86.2 ± 5.0						

^a^Subjects are recruited from the 31 patients who have developed scoliosis during the follow up period

Values are presented as mean ± standard deviation

Time 1 represents the time when scoliosis was first detected after loss of walking ability

Time 2 represents the time 1 year after the detection of scoliosis

Time 3 represents the time 2 years after the detection of scoliosis

### Scoliosis curve type changes

**Fig. 2:**
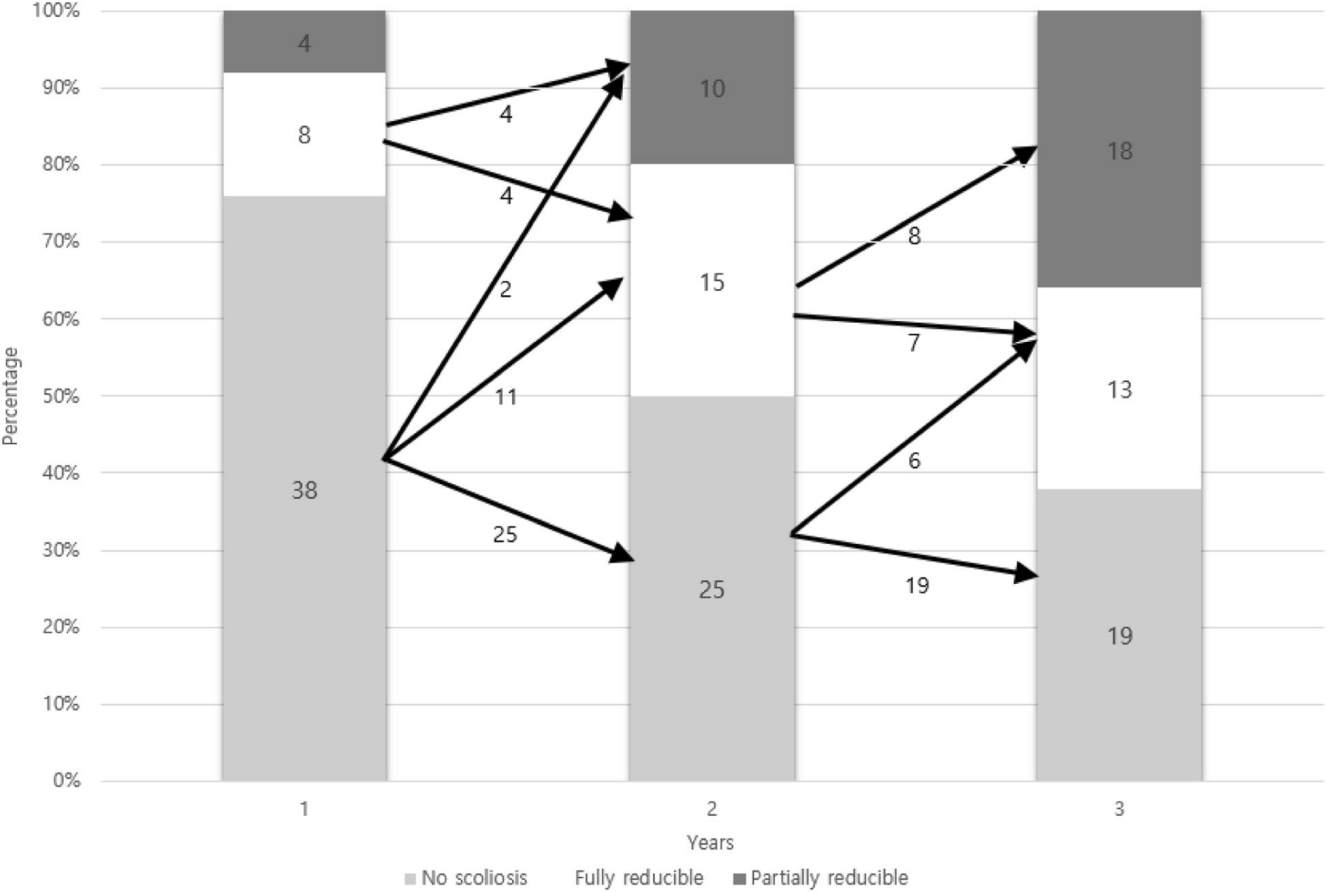
Values are presented as number of patients

Among the 31 boys who developed scoliosis, except for the 6 boys who developed scoliosis at the last year of follow-up and the 4 boys who already had nonstructural scoliosis at the first year of follow-up, only 2 boys had a course of “no scoliosis” that progressed directly to “nonstructural scoliosis.” The remaining 19 boys went through the sequence of (1) no scoliosis, (2) nonstructural scoliosis, and (3) structural scoliosis (Fig. 2).

### Changes in curve flexibility

Scoliosis was detected in the sitting position at the first visit after ambulation loss in 12 boys. In these 12 patients, consecutive follow-ups of supine and sitting radiographs were available for 2 years. Flexibility decreased over the follow-up period (*p* = 0.011). Mean values for flexibility were 75.5% at the first follow-up, 57.1% the next year, and 49.1% at the last follow-up. Cobb angle in this population increased over time (Table 2).

In the same context, for 25 patients whose scoliosis developed after 1 year follow up, consecutive follow-ups of supine and sitting radiographs were available for 2 time points. Flexibility decreased during follow-up period (*p* = 0.02). Mean values of flexibility were 60.7% at the first follow-up, 36.2% the next year. Cobb angle also increased over time in this group (Table 2).

### Correlation between spinal curve flexibility and other parameters

Spinal flexibility of 31 patients was inversely correlated with scoliosis curve angle in sitting and supine position (*r* = − 0.504 and *r* = − 0.774, respectively, *p* < 0.05 in both). Pulmonary function, forced vital capacity of % predicted was not correlated with spinal curve flexibility.

### Development of pelvic obliquity

There was no pelvic obliquity in the 19 boys who had not developed scoliosis. In the remaining 31 boys with scoliosis, 18 developed pelvic obliquity. In these boys, pelvic obliquity had an increasing tendency after ambulation loss both in the sitting and supine positions (Table 3). The Cobb angle in the sitting position had a significant correlation with pelvic obliquity both in sitting (r = 0.758, *p* = 0.000) and supine positions (r = 0.639, p = 0.000). The Cobb angle in the supine position also significantly correlated with pelvic obliquity both in the sitting (r = 0.844, p = 0.000) and supine positions (r = 0.810, p = 0.000). Pelvic obliquity in sitting and supine position did not show correlations with spinal curve flexibility.

**Table 3:** Pelvic obliquity of subjects

	Time 1			Time 2			Time 3		
Number of boys	Pelvic obliquity (°)		Flexibility (%)	Pelvic obliquity (°)		Flexibility (%)	Pelvic obliquity (°)		Flexibility (%)
	sitting	supine		sitting	supine		sitting	supine	
4	12.0 ± 8.0	5.3 ± 4.1	100.0 ± 40.0	11.4 ± 3.7	9.1 ± 3.5	27.3 ± 18.7	18.4 ± 7.3	12.6 ± 5.8	32.5 ± 12.7
10	9.1 ± 3.5	4.2 ± 4.9	61.5 ± 42.5	11.4 ± 1.1	6.9 ± 1.5	42.4 ± 10.7			
18	8.6 ± 1.0	2.5 ± 0.8	71.9 ± 9.2						

Values are presented as mean ± standard deviation

Time 1 represents the time when scoliosis was first detected after loss of walking ability

Time 2 represents the time 1 year after the detection of scoliosis

Time 3 represents the time 2 years after the detection of scoliosis

## Discussion

To the best of our knowledge, this is the first study to investigate curve flexibility in DMD patients. Curve flexibility has not been much investigated in neuromuscular patients due to relatively low incidence of the disease itself, and difficulties of regular visits due to physical disabilities of patients. However, the authors managed to gather information of DMD patients with regular follow ups with strict intervals, and adequate information of physical function and the timing of ambulation loss. We found that there is a period of fully reducible scoliosis curve soon after loss of walking ability in neuromuscular scoliosis of DMD patients. This is a period when spinal curve could be effectively reduced by spinal orthosis. Therefore, with early detection of scoliosis, and during the period when the curve is fully reducible, application of spinal braces should be considered.

Historically, bracing in neuromuscular scoliosis has been known to be ineffective [25–29]. Nevertheless, the beneficial effects of spinal bracing should be reconsidered in DMD patients for the following reasons: First, the exact timing of bracing was not indicated in existing studies [25, 27–29]; the subjects were those with scoliosis who rejected surgery, or who had low lung capacity and could not tolerate the surgery; this is thought to occur after rigid and structural scoliosis develop [29]. Second, studies on the effect of spinal bracing on scoliosis were written in the non-steroid era. Clearly, there has been a change in the course of scoliosis development in DMD patients who use steroids [4, 30, 31]. A number of studies have reported that glucocorticoids slowed the rate of curve progression [4, 30, 32]. Use of spinal braces in DMD patients who are taking glucocorticoids might further decrease the necessity for scoliosis surgery.

Etiology of spinal curve progression in neuromuscular conditions remains still only incompletely understood [33, 34]. Asymmetrically decreased tone of the paraspinal muscles is known to result in scoliotic curve formation, and it is expected to worsen in wheel chair bound patients. Loss of ambulation indicates that DMD has been already progressed to a certain degree that trunk muscles are also fairly impaired by then [27]. After loss of ambulation, spinal curvature rapidly progresses, and as it was shown in our study, flexibility also decreases almost simultaneously. It was contemplated that as the mechanical forces on the weaker side of spine are maintained, compensation builds on the skeletal system. As this condition continues, as long as the patients maintain sitting position in daily living, deformity of the musculoskeletal system may further progress, resulting in decreased spinal curve flexibility.

Within 2 years after loss of ambulation, scoliosis rapidly progressed that as many as half of patients who did not have rigid component of scoliosis eventually fell into the category of partially reducible scoliosis. Therefore, to detect scoliosis before rigid component of the curve develops, regular interval follow ups of at least within 1 year should be necessary. This is in line with guidelines of Birnkrant et al., that neuromuscular assessment and management should start in the early stage after loss of ambulation at least every 6 months [3, 5, 6]. Once the spinal curve becomes rigid, it is generally accepted that correction cannot be accomplished with orthotics. In such cases, surgical treatment might be needed. Nevertheless, surgery itself could be a burden for DMD patients because of progressive cardiomyopathy and respiratory muscle weakness [35].

In this study, authors evaluated scoliosis by assessing spine radiographs in two different positions; sitting and supine. There was a substantial difference in the Cobb angles based on the position. Therefore, it should be noted that scoliosis should be evaluated dynamically, and a single supine radiograph is not sufficient to diagnose the early phase of scoliosis development.

In the future, prospective studies, beginning orthotic management at very early phase of scoliosis development when scoliotic curve is still reducible, are necessary for providing evidences of the orthosis in preventing progression of neuromuscular scoliosis.

Neuromuscular scoliosis, unlike idiopathic scoliosis, is thought to be flaccid type and result in C-shaped curves [36]. This type of scoliosis extends its curve distally, causing pelvic obliquity [21]. Pelvic obliquity impairs sitting balance, hampering activities of daily living. Causes of pelvic obliquity are thought to include spinal deformities, hip contractures, leg-length discrepancy, or any combination of these factors [37]. Numerous studies reported that pelvic obliquity was more closely-related with spine deformity than with muscular imbalance below the pelvis [38]. Moreover, hip surgeries should have no effect on the correction of pelvic obliquity [38, 39]. The results of the present study are in line with those of previous studies. The subjects without scoliosis had no pelvic obliquity; in other words, only those with scoliosis developed pelvic obliquity. There was also a high correlation between Cobb angle and pelvic obliquity.

This study has several limitations. First, data collection was attempted with strict follow-up interval of 1 year, without missing radiographs. Therefore, the data were highly-refined, but there was a rather short follow-up of 2 years. More details regarding eventual courses of scoliosis curve and flexibility may be obtained with longer follow-up times and similar study designs. Second, Scoliosis consists of two components: lateral deviation and rotation. It is known that the apical vertebra is most deeply rotated [39]. In the present study, however, only lateral deviation was considered for evaluation. We evaluated the lateral deviation because the participants in were in the early stage of scoliosis, only 2 years after ambulation loss. Because the degree of rotation increases according to the severity of coronary curves, we assumed that the rotative degree would not have a serious impact [40, 41]. Lastly, the participants are grouped according to the yearly time-frame after loss of ambulation, not according to the magnitude of the spinal curve. Flexibilities of curves are largely influenced by the degree of the curve. However, in our study, as it is shown in Table 1, Cobb angle of standard deviation of each group is around 5 degrees, which means that within each group, the degree of Cobb angel is relatively homogenous. Also, Cheung et al. reported that in adolescent idiopathic scoliosis, curve flexibility is the only parameter that significantly influences in-brace correction in adolescent idiopathic scoliosis, regardless of curve size or age [42].

## Conclusion

In this study, after loss of ambulation when scoliosis starts to develop, there is a period of fully reducible curve in DMD scoliosis patients. This result suggests that in the early stage of scoliosis, wherein flexibility is maintained without structural scoliosis, interventions such as bracings should be considered in DMD scoliosis.

Also, scoliosis curve in DMD patients should be evaluated dynamically to detect the scoliosis when the curve is fully reducible. This study could be a cornerstone for further studies involving application of spinal braces for neuromuscular scoliosis.

## Data Availability

This article is a retrospective study, and the available data were collected from 50 DMD patients and listed in the tables. However, because we plan a further study of the patient group with orthotic treatment, we do not wish to share the raw data at present. The datasets used and/or analyzed during the current study are available from the corresponding author on reasonable request.

## Abbreviations

DMD: Duchenne Muscular Scoliosis
